# Clinical implementation of a multifaceted quality assurance phantom for high-precision radiation therapy: an institutional experience

**DOI:** 10.1186/s43046-025-00314-x

**Published:** 2025-09-01

**Authors:** Sandeep Singh, Abhay Kumar Singh, Dipesh, Manindra Bhushan, Supratik Sen, Mahipal, Raj Pal Singh, Anuj Vijay, Munish Gairola

**Affiliations:** 1 Department of Radiation Oncology, Division of Medical Physics, Rajiv Gandhi Cancer Institute and Research Center, New Delhi, New Delhi, India; 2 Department of Physics, GLA University, Mathura, Uttar Pradesh,, Mathura, India; 3 Department of Radiation Oncology, Division of Medical Physics, Rajiv Gandhi Cancer Institute and Research Center, New Delhi, New Delhi, India; 4 Department of Radiation Oncology, Rajiv Gandhi Cancer Institute and Research Center, New Delhi, New Delhi, India

**Keywords:** Stereotactic radiosurgery (SRS) quality assurance, End-to-end testing in radiotherapy, Isocenter verification, Patient positioning accuracy, Phantom-based dosimetric validation

## Abstract

**Aim:**

This study aimed to evaluate the multifaceted clinical utility of the RUBY phantom as a comprehensive quality assurance (QA) platform in high-precision radiotherapy, particularly for stereotactic radiosurgery (SRS) and stereotactic radiotherapy (SRT). The objective was to validate its performance in patient positioning, imaging system accuracy, isocenter congruency, and treatment plan verification across various complex clinical scenarios.

**Materials and methods:**

A series of QA workflows were conducted using the RUBY phantom and its dedicated modular inserts. These included CBCT and MV/kV planar imaging for patient alignment, the Winston-Lutz insert for isocenter verification, end-to-end testing for full-chain validation, and multi-metastasis and patient-specific inserts for point dose verification. Plans were created in Eclipse TPS (v15.6) and delivered using a Varian TrueBeam STx linear accelerator with high-definition MLC. PTW Semiflex 3D and PinPoint 3D ionization chambers were used for all dosimetric verifications.

**Results:**

CBCT- and planar image-guided alignments showed sub-millimetric deviations, with post-correction alignment verified through coincident laser and surface markers. Winston-Lutz testing across various angles demonstrated maximal deviations of ≤ 0.4 mm, which was within TG-142 recommendations. Point dose measurements for 61 SRS plans showed agreement within ± 3% of TPS calculations. End-to-end testing revealed dose discrepancies of < 1% in both coplanar and non-coplanar beam arrangements. Multi-target plans using single- and multi-isocenter approaches showed deviations of − 0.23% to − 0.50%, confirming excellent dosimetric and geometric accuracy.

**Conclusion:**

The RUBY phantom demonstrated exceptional precision, reproducibility, and clinical adaptability across a spectrum of advanced radiotherapy QA tasks. Its integration enables the end-to-end validation of modern treatment protocols, ensuring alignment, imaging, and accuracy of dose delivery. These findings establish the RUBY phantom as a cornerstone QA solution for enhancing safety, efficacy, and reliability in high-precision radiotherapy.

**Supplementary information:**

The online version contains supplementary material available at 10.1186/s43046-025-00314-x.

## Introduction

The evolution of high-precision radiotherapy has heralded a new era in cancer treatment, prioritizing targeted tumour eradication while minimizing harm to surrounding healthy tissues. This paradigm shift necessitates a meticulous approach to treatment delivery, where the accuracy of patient positioning and the reliability of imaging systems are paramount [1]. Even the slightest deviation in alignment or dose calculation can significantly impact treatment efficacy and patient safety. Addressing these challenges requires sophisticated technology and rigorous quality assurance (QA) processes to validate and enhance clinical workflows [2]. In this context, the RUBY phantom (PTW, Freiburg, Germany) emerges as a versatile and indispensable tool for ensuring the integrity of radiotherapy systems and workflows. The RUBY phantom has been engineered to simulate complex clinical scenarios, enabling comprehensive testing and calibration of these systems [3, 4]. Its innovative design and modular inserts support a wide range of QA protocols, from verifying the alignment of treatment isocenters to assessing the performance of imaging systems and treatment planning software. One of the key applications is its role in patient positioning QA, as accurate positioning is critical for maximizing the therapeutic benefit of radiotherapy while reducing the risk of radiation-induced complications [5]. It facilitates the evaluation of patient localization systems by enabling clinicians to simulate translational and rotational misalignments. This ensures that image registration algorithms and couch correction mechanisms function seamlessly, maintaining alignment accuracy across all treatment fractions. In addition to patient positioning, it verifies the congruence of imaging and treatment isocenters—a fundamental requirement for effective image-guided radiotherapy (IGRT). Its Linac QA insert is specifically designed to perform Winston-Lutz tests, a standard QA procedure for evaluating the precision of imaging systems in aligning with the linear accelerators (linac) beamline [6, 7]. The integration of a ceramic ball within the phantom allows for precise measurement of isocenter deviations, ensuring that imaging systems such as CBCT and planar kV/MV imaging consistently align with the treatment beam. Beyond imaging and positioning, it plays a pivotal role in treatment plan verification, addressing both dosimetric and geometric aspects of radiotherapy. Its compatibility with various detectors, including ionization chambers and radiochromic films, enables it to perform both point-dose and two-dimensional dose verifications with high precision. This is particularly beneficial for complex treatment techniques, such as stereotactic radiotherapy (SRT) and single-isocenter multi-metastasis treatments, where dose delivery accuracy is critical. The phantom's ability to support non-coplanar beam arrangements without repositioning further enhances its utility in verifying advanced treatment plans [8–10].

The applications of phantom extend beyond individual QA tasks to encompass comprehensive end-to-end testing [11, 12]. Its System QA insert integrates tissue-equivalent materials of varying densities (Lung, brain, and bone) and MRI-visible structures, enabling realistic simulation of patient anatomy and facilitating multimodal imaging registration tests. These features allow evaluation of the entire treatment chain, from planning CT and MRI image acquisition to treatment delivery, ensuring that each step meets the highest standards of precision and accuracy. Furthermore, the phantom is designed to adapt to emerging trends in radiotherapy, such as Surface-Guided Radiotherapy (SGRT) and mask-based immobilization techniques. Its compatibility with SGRT systems, including Vision-RT and C-RAD, ensures accurate surface detection and alignment, while its integration with mask systems such as Brainlab and CQ Medical facilitates QA workflows for head and neck treatments. These capabilities make this an invaluable asset in supporting the transition to more personalized and patient-centric radiotherapy approaches.

This manuscript aims to provide a comprehensive overview of the RUBY phantom’s capabilities, focusing on its contributions to enhancing QA protocols in high-precision radiotherapy. We highlight the phantom’s role in bridging the gap between theoretical advancements and clinical practice by exploring its applications in patient positioning, imaging system calibration, dose verification, and end-to-end testing. As radiotherapy continues evolving, reliable QA tools are becoming increasingly critical. By ensuring the accuracy and consistency of radiotherapy workflows, this phantom ultimately contributes to improved patient outcomes, reinforcing its status as a cornerstone of modern cancer treatment.

## Materials and methods

This study evaluated the RUBY phantom’s multifaceted capabilities in ensuring quality assurance (QA) for high-precision radiotherapy. Specifically, the study examined its applications in validating patient positioning accuracy, calibrating imaging systems, verifying treatment plans, and performing comprehensive end-to-end tests. These workflows were analyzed under clinically relevant conditions using state-of-the-art imaging, planning, and treatment platforms. These investigations aimed to validate this phantom robustly as a critical tool for radiotherapy QA, ensuring precision and consistency in clinical applications.

Our institute has various phantom inserts tailored for comprehensive quality assurance and imaging validation tasks. These include (1) CBCT and MV/kV planar imaging inserts with precisely defined displacements and a tilted base for geometric accuracy checks and for Winston-Lutz test (isocenter verification); (2) an end-to-end testing QA insert for simulating the complete treatment workflow; (3) a patient-specific point dose measurement tool for in vivo or phantom-based dosimetry; and (4) a multiple metastasis application insert designed for evaluating multi-target stereotactic treatment planning and delivery accuracy. The different chambers used were PTW Semiflex 3D (Sensitive volume 0.07 cm^3^) (T31010) (PTW, Freiburg, Germany) and PTW PinPoint 3D (Sensitive volume 0.016 cm^3^) (T31022) (PTW, Freiburg, Germany). The study was conducted on a Varian TrueBeam STx C-arm linear accelerator (Varian Medical Systems, Palo Alto, CA, USA), an upgrade package on the TrueBeam, which is designed to enhance stereotactic radiotherapy (SRT) and stereotactic body radiation therapy (SBRT) capabilities. The system has a high-definition multileaf collimator (HD MLC) featuring 2.5 mm leaf widths, allowing for highly conformal dose distributions to small and irregularly shaped targets. It supports high-dose rate delivery and integrates onboard imaging modalities, including cone-beam CT, to facilitate accurate patient positioning and adaptive treatment workflows. Treatment planning and contouring for this study were performed using the Eclipse Treatment Planning System (version 15.6, Varian Medical Systems, Palo Alto, CA, USA) using 6 MV FFF (Flattening filter free) energy and dose rate of 1400 MU/min (Monitor Units/Minute).

### Simulation

**Fig. 1:**
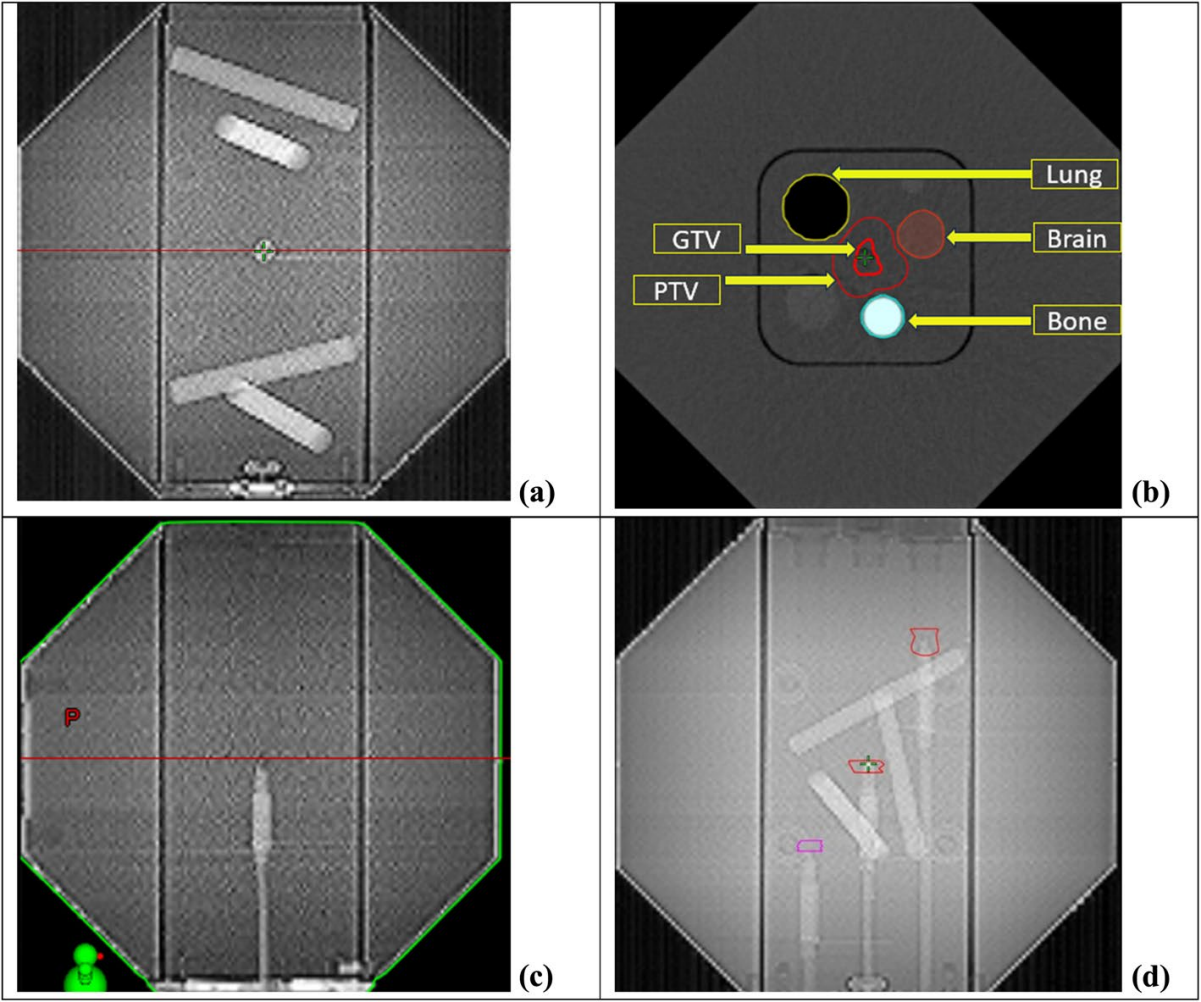
The figure represents the digitally reconstructed radiograph (DRR) of different inserts, where **a** shows the inserts used in CBCT, MV/KV planar dose verification and for Winston-Lutz Test, **b** shows the end-to-end test insert, **c** shows the patient-specific point dose verification with chamber, and **d** shows the multi-metastasis insert showing embedded ion chamber positions corresponding to three distinct planning target volumes (PTVs). The contours outlined in red, green, and magenta represent the PTVs defined for multi-target stereotactic radiotherapy. Dose measurements were performed at the geometric centers of these PTVs to ensure sampling from the high-dose region and avoid dose gradient artifacts

CT simulation was performed for each phantom insert to replicate clinically relevant imaging and treatment workflows. For the CBCT and MV/kV planar imaging insert, the phantom was positioned without any predefined displacement and tilted base to assess image registration accuracy across modalities for patient position verification. The Winston-Lutz testing insert was scanned in a neutral position to establish the spatial relationship between the radiation isocenter and mechanical components, aiding in isocenter localization during treatment planning. The end-to-end testing QA insert underwent full CT simulation, including immobilization setup, enabling verification of the entire radiotherapy chain—from imaging and planning to dose delivery. The insert was scanned with the embedded ion chamber or diode at the reference position for patient-specific point dose measurement, ensuring accurate dose calculation and later measurement validation. Lastly, the multiple metastasis application insert was scanned with three embedded ionization chambers to evaluate multi-lesion stereotactic treatment planning, focusing on geometric precision and dosimetric accuracy across multiple isocenters or a single isocenter approach (Fig. 1). All scans were acquired using Siemens Somatom go.Sim CT simulator (Siemens Healthineers, Germany), with 1 mm slice thickness and field of view optimized based on the insert type and clinical objective.

#### Patient positioning QA

Patient positioning accuracy was assessed using the RUBY phantom under controlled misalignment conditions. After acquiring planning CT, the phantom was placed on the treatment couch with intentional translational and rotational offsets. These were introduced via the phantom’s grey/red lines and tilting base. CBCT or planar imaging was used to detect displacements, and automatic couch corrections were applied. Visual alignment was verified using the black surface lines and room lasers. Detected shifts were recorded and analyzed against predefined reference values. Similarly, planar imaging systems were tested by acquiring kV and MV images, and manual and automated registration methods were evaluated.

### Winston-Lutz test

The Winston-Lutz (WL) test is a fundamental quality assurance procedure in stereotactic radiosurgery (SRS) and stereotactic radiotherapy (SRT), designed to verify the congruence of the mechanical, imaging, and radiation isocenters of a linear accelerator. Precise isocenter alignment is critical for achieving sub-millimetric accuracy in high-dose, small-volume treatments. This study performed the WL test using the RUBY phantom equipped with a dedicated Winston-Lutz insert containing a centrally embedded ceramic ball as a radiopaque marker. The phantom was initially aligned using room lasers to establish a reference setup. MV portal images were acquired at multiple gantry (0°, 45°, 90°, 135°, 180°, 225°, 270°, 315°), collimator (0°, 45°, 90°, 135°, 180°, 225°, 270°, 315°), and couch angles (0°, 30°, 45°, 90°, 270°, 315°, 330°), simulating diverse treatment geometries. These images were subsequently analyzed using the Offline Review module in the Varian Eclipse treatment planning system (TPS).

#### Patient-specific dosimetric QA

Point dose verification was performed for 61 stereotactic radiosurgery (SRS) patient plans using PTW Semiflex 3D and PTW PinPoint 3D ionization chambers. The range of plan types, number of targets, prescription doses, and anatomical regions covered was outlined in Table 1. Each clinical treatment plan was transferred from the TPS and recalculated on a dedicated QA phantom to ensure accurate dose delivery under measurement conditions. The PTW detectors were securely positioned in the phantom’s Patient QA insert using dedicated holders, allowing for precise alignment with the planned isocenter. The isocenter dose from the TPS was used as the reference calculated dose. QA plans were subsequently delivered to the phantom, and point dose measurements were acquired at the isocenter using both detectors. All measurements were corrected for temperature and pressure in accordance with standard protocols. The percentage difference between the TPS-calculated and measured doses was then computed for each plan to assess dosimetric accuracy.

**Table 1:** The key characteristics of the 61 stereotactic radiosurgery (SRS) plans evaluated during end-to-end point dose verification

Parameter	Range/distribution
Number of targets	1–5
Target location(s)	Brain (frontal, parietal, cerebellum), skull base, brainstem-adjacent
Prescribed dose per target	12–24 Gy
Number of arcs	1–5
Technique	VMAT
Couch angles used	0°, 30°, 330°
Plan type	Single-target (*n* = 25), multi-target (*n* = 36)
PTV volume range	0.15 cc–24.0 cc
OAR constraints used	Brainstem, optic chiasm, optic nerves, cochlea (where applicable)
Dose gradient complexity	Mild (85–90% prescription isodose line) to steep (70–75% prescription isodose line)

The dataset includes both single-target and multi-target plans with varying anatomical locations, prescription doses, and planning complexities. All plans were recalculated on the QA phantom, and point dose measurements were performed at the planned isocenter. This table highlights the diversity of the test cohort, supporting the generalizability and clinical relevance of the dosimetric validation.

### End-to-end testing

End-to-end testing was performed to validate the complete radiotherapy workflow, from imaging and treatment planning to dose delivery. A planning CT dataset of the phantom was acquired, with the System QA insert providing realistic tissue-equivalent simulation. The dataset was imported into the TPS to evaluate image quality, delineation, and treatment planning, as shown in Fig. 1b. The point dose verification was performed for four planning configurations: coplanar, non-coplanar, coplanar with tilted base, and non-coplanar with tilted base. These scenarios were designed to simulate realistic clinical setups and evaluate the integrity of the entire treatment workflow. All plans used for end-to-end testing and multi-metastasis verification were developed following our institutional stereotactic radiotherapy planning protocol, aligned with AAPM TG-101 guidelines [13]. Each target received a prescription dose of 5 Gy per fraction, with at least 95% of the planning target volume (PTV) covered by 100% of the prescribed dose, typically normalized to the 80–85% isodose line. The PTVs were generated by isotopically expanding the gross tumor volume (GTV) by 1 mm. Coplanar plans consisted of two full volumetric modulated arcs with collimator angles between 30° and 45°, while non-coplanar plans included two full arcs and two partial arcs, employing couch angles of 30° and 330°, with gantry arcs spanning approximately 181°–30° to avoid collisions. Although the phantom lacks anatomical organs-at-risk (OARs), simulated dose constraints were applied consistent with cranial SRS planning standards, such as limiting the maximum dose to surrounding material to < 3 Gy to 0.1 cc, targeting a conformity index (CI) of 1.0–1.2. Dose calculations were performed using the Acuros XB algorithm (v15.6) with heterogeneity correction enabled and a grid size of 1 mm.

### Multi metastasis QA

**Fig. 2:**
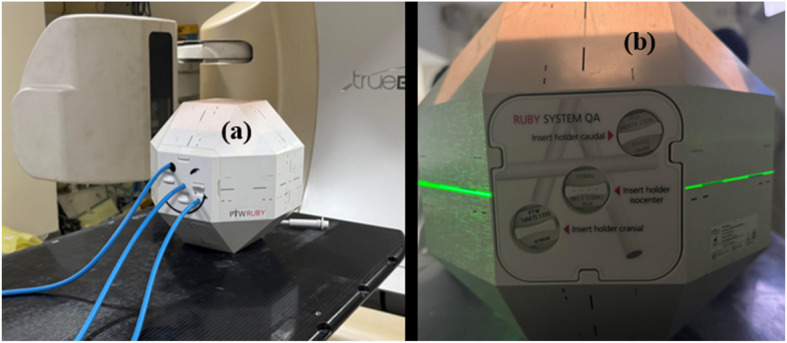
**7a** RUBY with chamber. **7b** Position of three different locations of chamber

**Fig. 3:**
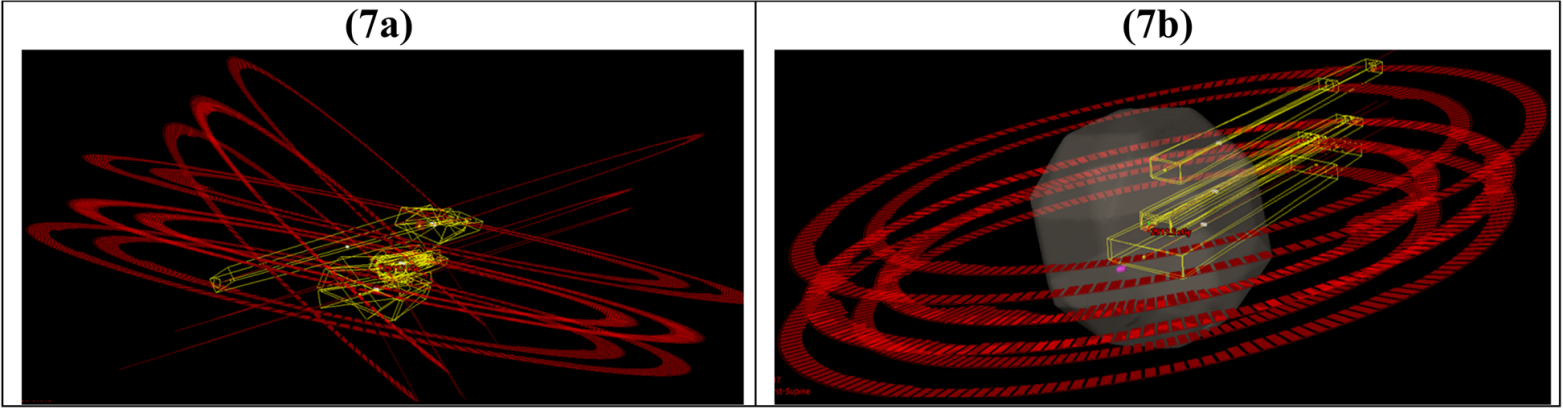
The plans for multi-metastasis insert. **8a** shows the multi-isocenter non-coplanar (MINC), and **8b** shows the multi-isocenter coplanar (MIC) plans

**Fig. 4:**
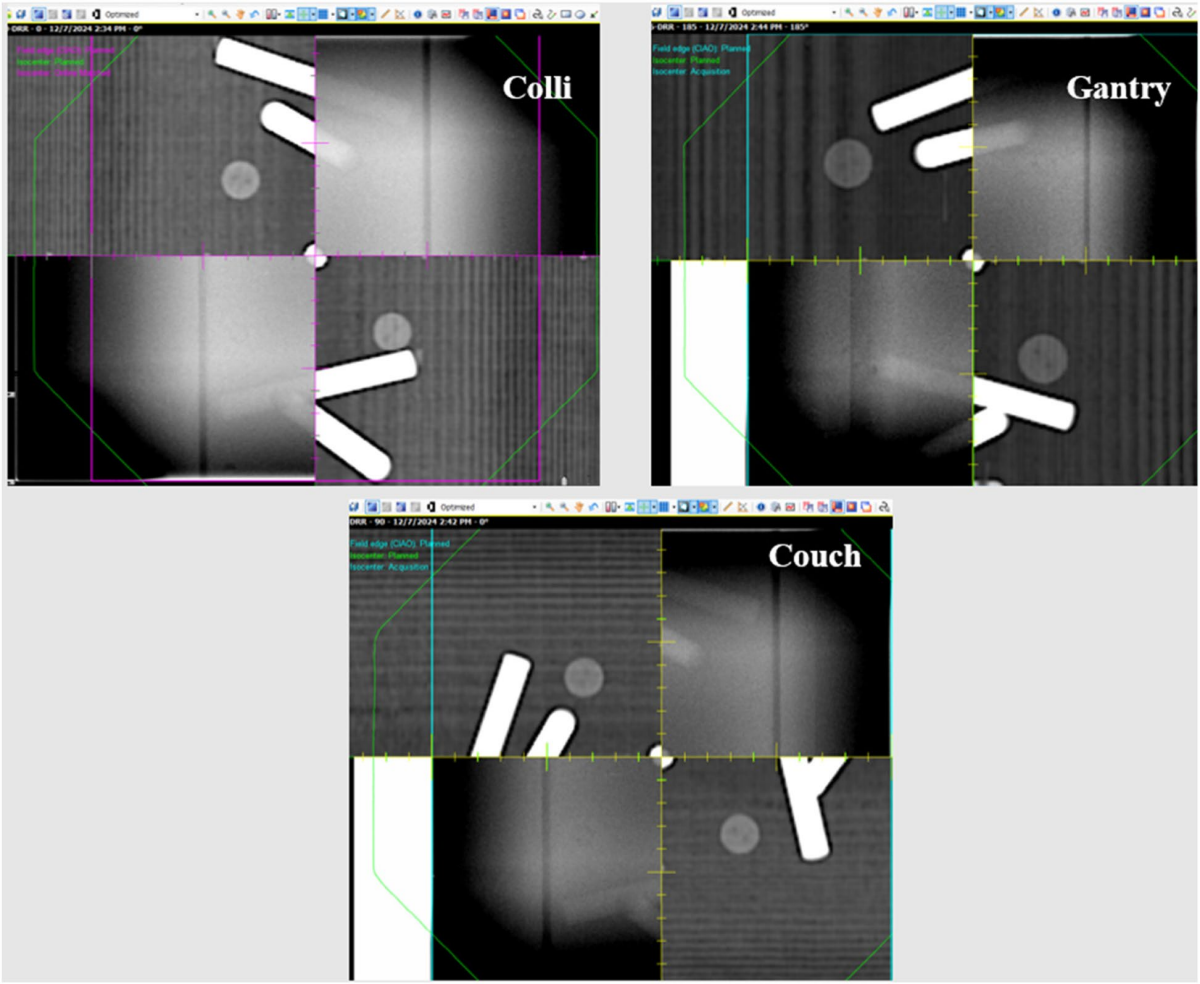
Analysis of Winston-Lutz test (matching of MV image with DRR generated from the planning CT)

Point dose verification was conducted using the multi-metastasis insert equipped with three predefined chamber locations to simulate multiple lesion sites, as shown in Fig. 2. A planning CT dataset of the phantom was acquired and imported into the treatment planning system (TPS) to create single-isocenter and multi-isocenter stereotactic treatment plans, incorporating coplanar and non-coplanar beam arrangements. The plans were recalculated on the phantom dataset, and dose delivery was performed accordingly. Ionization chambers were positioned sequentially at each chamber location, and measured doses were compared with TPS-calculated values to verify the dosimetric accuracy of complex stereotactic treatment strategies involving multiple targets. Four distinct plan (5 Gy dose) configurations as shown in Table 2 were created to simulate various clinical scenarios: (1) single isocenter coplanar (SIC), (2) single isocenter non-coplanar (SINC), (3) multi-isocenter coplanar (MIC) (Fig. 3a), and (4) multi-isocenter non-coplanar (MINC) (Fig. 3b).

**Table 2:** The setup used for different plans in multi metastasis QA

Plan name	Number of isocenters	Couch angle	Number of arcs without couch angle	Number of arcs without couch angle
Single isocenter coplanar (SIC)	1	–	2 full arcs	–
Single isocenter non-coplanar (SINC)	1	30° and 330^°^	2 full arcs	2 partial arcs (181^°^–30° CW* and 179^°^–330° CCW**)
Multi-isocenter coplanar (MIC)	3	–	6 full arcs (2 arcs per isocenter)	–
Multi-isocenter non-coplanar (MINC)	3	30° and 330^°^	6 full arcs (2 arcs per isocenter)	2 partial arcs (181^°^–30° CW* and 179^°^–330° CCW**)

*Clockwise

**Counter-clock wise

## Results

### CBCT and MV/KV planar imaging

Patient positioning verification using CBCT imaging was conducted with standard clinical parameters to assess the system’s ability to detect and correct misalignments through automated couch shifts. Accurate image registration between the CBCT and planning CT datasets was achieved using four distinct bony landmarks within the phantom. Initial alignment was performed by matching the phantom’s surface lasers with the green line (without the tilted base) or the red line (with the tilted base); after automatic couch correction, the black alignment line precisely coincided with the room lasers, confirming successful registration and system alignment. System-computed displacement values were compared against the predefined tolerance thresholds specified for the phantom, with all deviations falling within acceptable limits. The high contrast of internal bone structures on both kV and MV planar images enabled consistent manual and automated registration with digitally reconstructed radiographs (DRRs). Displacement data were systematically recorded for QA documentation and trend analysis. Overall, the results confirmed high geometric fidelity and correction accuracy in CBCT-guided patient positioning. The results are shown in Table 3 below.

**Table 3:** The observed shift using CBCT and 2D planar imaging

Matched with grayline (without tilted base)				Matched with red line (with titled base)		
	Baseline	Measured		Baseline	Measured	
		CBCT	MV/KV		CBCT	MV/KV
Vrt (cm)	− 1.80	− 1.7	− 1.7	− 1.20	− 1.1	− 1.1
Lng (cm)	− 1.40	− 1.3	− 1.3	− 1.00	− 0.9	− 0.9
Lat (cm)	2.50	2.4	2.4	1.50	1.4	1.5
Rtn (^o^)				1.00	0.9	0.9

### Winston-Lutz test

The acquired MV images were analyzed using the Offline Review module of the Varian Eclipse treatment planning system (TPS). Each image was compared against the corresponding digitally reconstructed radiograph (DRR) derived from the planning CT to evaluate any positional deviation of the ceramic ball relative to the center of the radiation field (Fig. 4). The test was repeated multiple times, and the vertical, longitudinal, and lateral displacements were recorded and evaluated against the tolerance criteria, which is a maximum deviation of ≤ 1 mm in any direction [14–16].

**Fig. 5:**
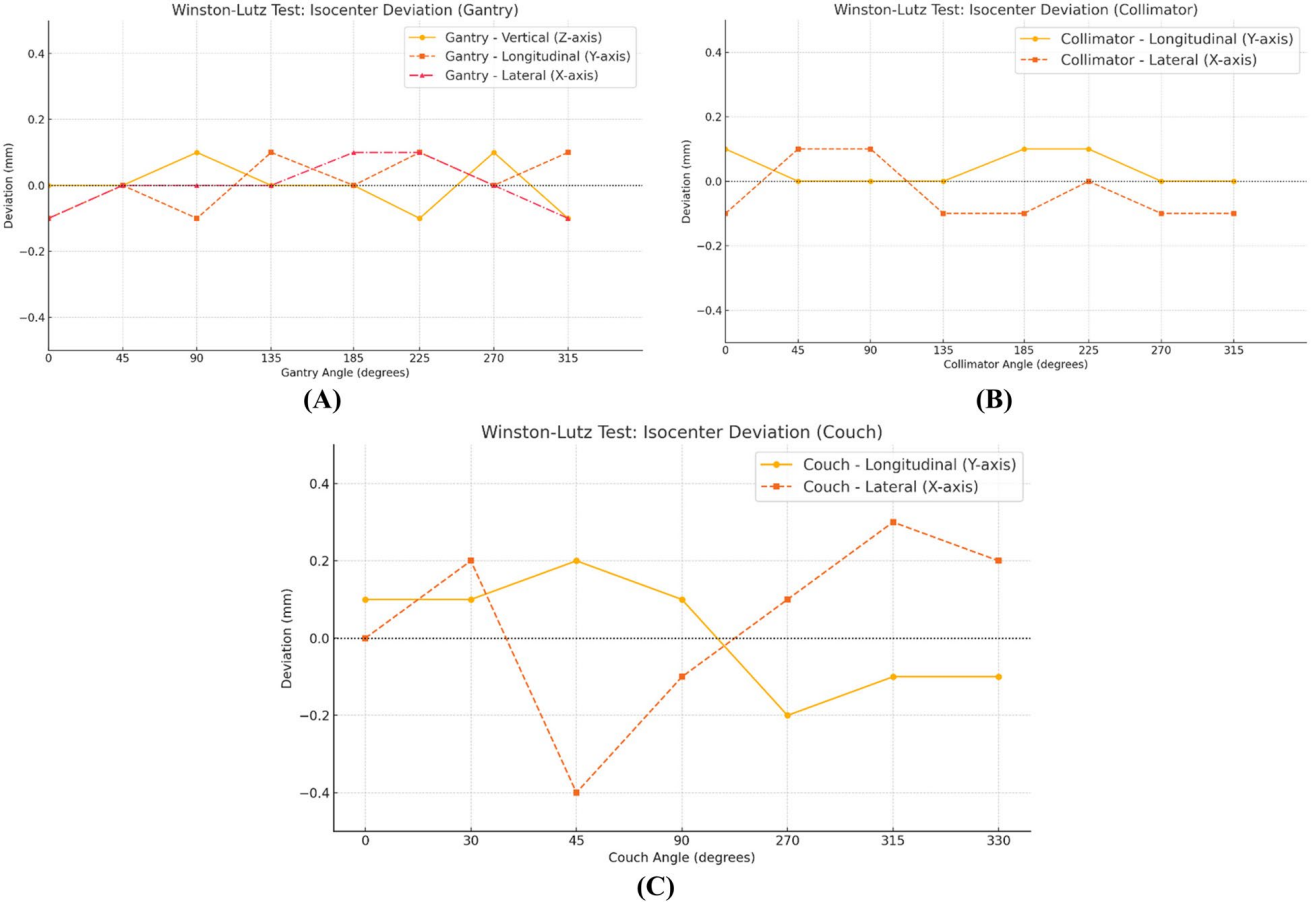
The results of the Winston-Lutz test for gantry (**A**), collimator (**B**), and couch (**C**)

Gantry angle variations resulted in minimal positional deviations, with all values within ± 0.1 mm. Notable deviations included + 0.1 mm along the z-axis (IEC Transverse) at 90° and 270°, and + 0.1 mm along the *x*-axis at 185° and 225°. The most significant y-axis deviation was + 0.1 mm at 135°, 225°, and 315°, all well within the 1 mm tolerance (Fig. 5A). For collimator rotations (0° to 315°), all measured deviations were within ± 0.1 mm across all axes. The largest observed displacements occurred at 90° and 45°, with deviations of + 0.1 mm in the *x*-axis (IEC lateral). The maximum deviation in the y-axis (IEC longitudinal) was + 0.1 mm, indicating excellent rotational precision of the collimator system (Fig. 5B). Couch rotations produced slightly larger deviations compared to gantry and collimator tests. The maximum displacement was observed at 45°, with a *y*-axis deviation of + 0.2 mm and an x-axis deviation of − 0.4 mm. An additional *x*-axis shift of + 0.3 mm was noted at 315°. Despite these comparatively larger deviations, all values remained within the TG-142 [17–19] tolerance threshold of 1 mm, as illustrated in (Fig. 5C).

### Patient-specific point dose verification

**Fig. 6:**
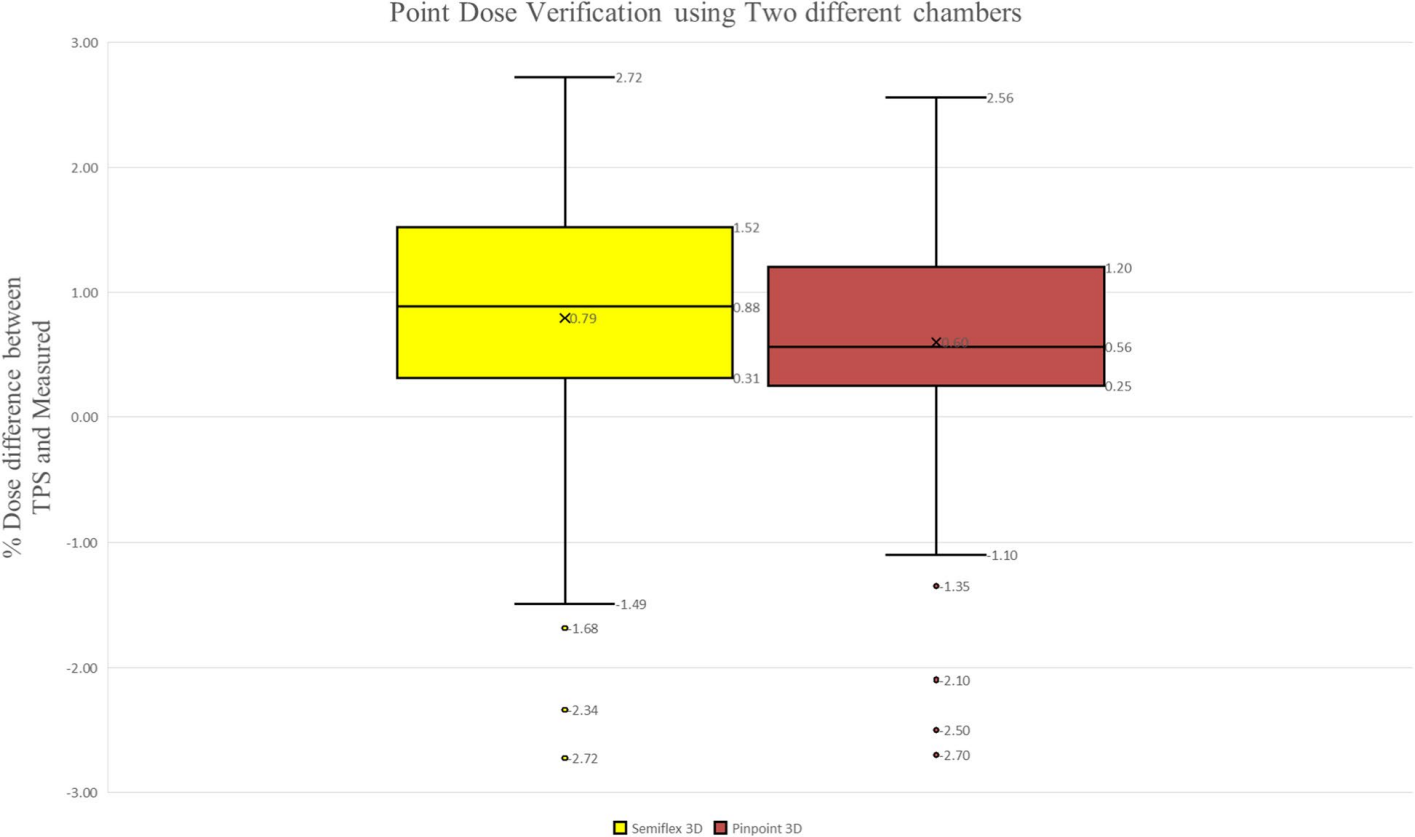
The point dose deviation between TPS calculated and chamber measured using Semiflex 3D chamber and PinPoint 3D chamber using RUBY Phantom

Based on the analysis of 61 paired point dose measurements comparing TPS-calculated doses with those measured using Semiflex 3D and Pinpoint 3D ionization chambers, a consistent trend of slight TPS overestimation was observed. The median percentage dose differences were + 0.79% for Semiflex 3D and + 0.60% for Pinpoint 3D, with all values falling within the clinically acceptable ± 3% [20–22] range. The majority of readings for both chambers indicated positive deviations, suggesting that the TPS-calculated dose was generally higher than the measured dose. The maximum positive deviation reached + 2.72% for Semiflex and + 2.56% for Pinpoint, while the most negative values were − 2.72% and − 2.70%, respectively. This systematic positive bias could be attributed to factors such as volume averaging effects, minor detector positioning inaccuracies, and inherent limitations in TPS beam modeling, particularly in complex, high-gradient regions. Importantly, both chambers demonstrated good agreement with TPS, validating their suitability for stereotactic point dose verification in routine clinical practice (Fig. 6).

### End-to-end testing

**Fig. 7:**
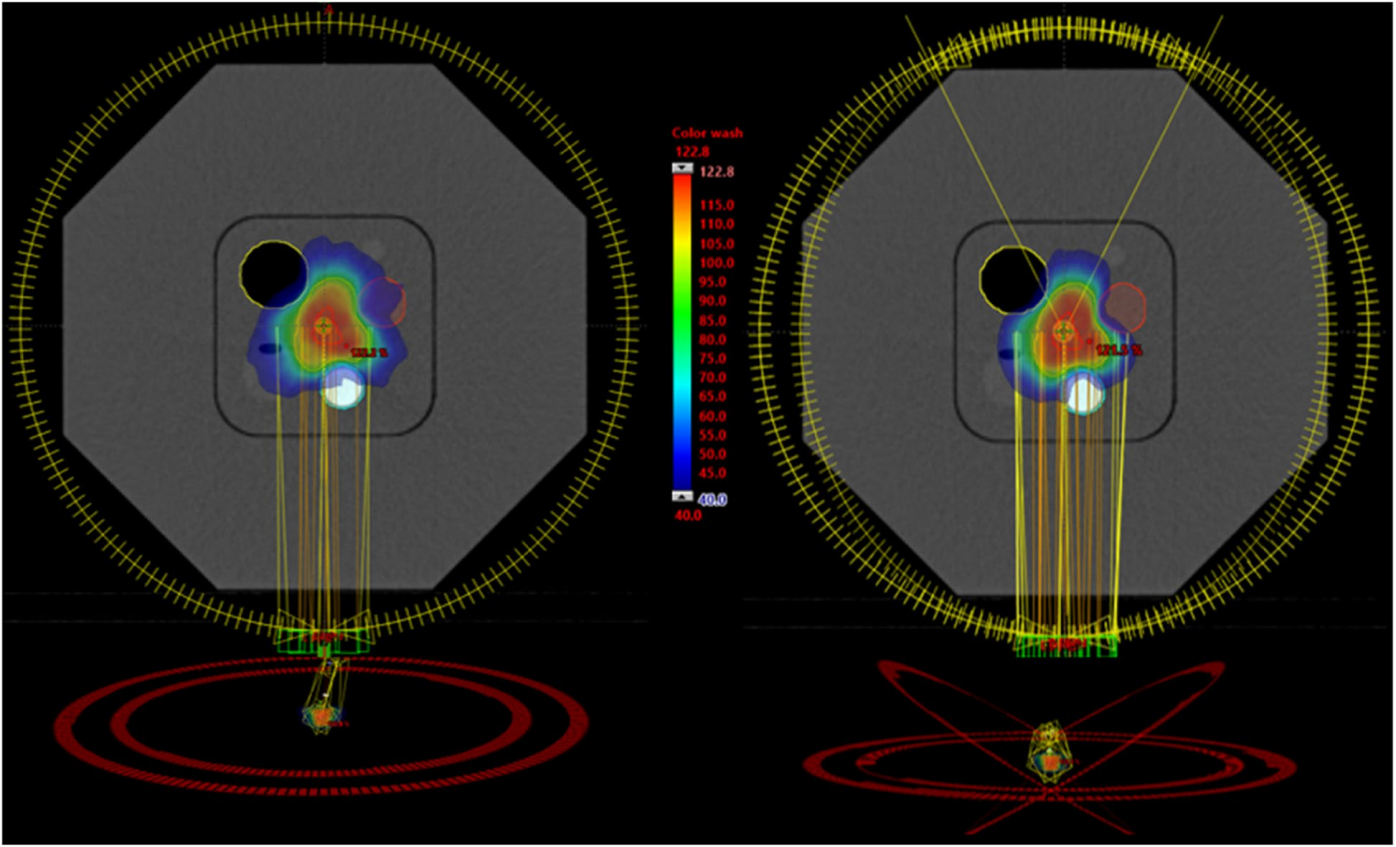
The end-to-end test plans using coplanar and non-coplanar beam arrangement

The surrounding structures around the PTV were air, high-density material, and soft tissue material, which is equivalent to the lung, bone, and brain tissue in the human body (as per ICRU-44/−46 standards) [23, 24]. We had observed that there is very little difference between the standard value and measured values of HU, density, diameter, and volumes of structures. The System QA insert enables the integration of the patient positioning workflows in the end-to-end test chain. The insert is visible on CBCT imaging, enabling a validation of the image-guided patient positioning workflow. The plan setup was shown in Fig. 7. The TPS-calculated isocenter doses ranged from 550.5 to 589.5 cGy, while the corresponding measured doses varied between 544.9 cGy and 585.7 cGy. The absolute dose deviations were 0.65% for the coplanar plan, 0.95% for the non-coplanar plan, 1.02% for the coplanar with tilted base, and 0.98% for the non-coplanar with tilted base. Notably, the inclusion of a tilted base slightly increased the deviation, likely due to minor setup uncertainties and altered beam geometries. However, all deviations remained within the ± 3% tolerance recommended for stereotactic QA, confirming the robustness and geometric fidelity of the treatment system across varying plan complexities and couch configurations (Table 4).

**Table 4:** The end-to-end test results for plan setups

Plan name	TPS (cGy)	Measured (cGy)	Deviation (%)
Coplanar	589.5	585.7	0.65
Non-coplanar	570.4	565.0	0.95
Coplanar with titled base	550.5	544.9	1.02
Non-coplanar with tilted base	560.4	585	0.98

### Multi-metastasis QA

The RUBY phantom was utilized comprehensively to verify the accuracy of SRS/SRT treatment plans involving multiple target volumes. This insert accommodates the placement of three detectors at distinct spatial positions, each identifiable via embedded CT markers. The configuration allows for a maximum longitudinal separation of 10 cm, lateral spacing of up to 5.5 cm, and a vertical offset of 4.8 cm between detectors. As such, the arrangement emulates the anatomical distribution of three intracranial metastases undergoing simultaneous treatment. All plans were delivered on the treatment machine, and point dose verification was performed for the above four plans. The comparison between the TPS-calculated doses and the measured values using a high-precision ionization chamber revealed excellent agreement across all plans (Table 5). The percentage deviations between the TPS and measured doses were within clinically acceptable limits (± 3%). Specifically, the SIC plan showed a deviation of − 0.23%, while the SINC plan exhibited a slightly higher deviation of − 0.50%. The MIC and MINC plans demonstrated deviations of − 0.43% and − 0.22% for multi-isocenter scenarios, respectively. These findings confirm the dosimetric accuracy and consistency of both coplanar and non-coplanar stereotactic treatment deliveries, regardless of the number of isocenters involved.

**Table 5:** The point dose deviation between TPS versus those measured for a different plan

Plan name	TPS	Measured	% Deviation
SIC	586.4	587.8	− 0.23
SINC	575.8	578	− 0.50
MIC	566.8	569.2	− 0.43
MINC	565.0	566.2	− 0.22

## Discussion

### Comparison with established SRS QA phantoms

The RUBY modular phantom offers a multifaceted approach to stereotactic QA, making it instructive to compare it against other widely used systems such as the Lucy 3D QA Phantom, Sun Nuclear’s SRS MapCheck (with StereoPHAN), and the QUASAR™ Penta-Guide. The Lucy phantom has long been regarded as a gold standard for SRS/SRT QA, with sub-millimeter manufacturing tolerances (\ ~ 0.1 mm) that meet the stringent demands of high-precision treatments. Like the RUBY, Lucy [25] is a modular system supporting various inserts for end-to-end testing, including image fusion accuracy, point-dose measurement, and film dosimetry. RUBY similarly emphasizes versatility, consolidating many of these functions into a single system: alignment checks, CT–MR fusion assessment, point-dose measurement, and film or detector inserts. Functionally, RUBY and Lucy are comparably versatile, both supporting end-to-end evaluations of isocenter localization, imaging alignment, and dose delivery.

In contrast, Sun Nuclear’s SRS MapCheck [26, 27] is specialized for high-resolution planar dose verification. When embedded within the StereoPHAN, it streamlines patient-specific QA by providing immediate 2D dose distributions, eliminating the need for film development. This makes SRS MapCheck highly efficient for routine plan verification, reducing QA time from hours to minutes. However, its focused application comes at the cost of broader functionality—it does not inherently support imaging system checks or multimodality alignment, but it can be used for assessment of IGRT system [28, 29]. The QUASAR™ Penta-Guide [4], meanwhile, is specifically tailored to IGRT geometry QA. Globally recognized for daily image-guidance checks, it ensures the alignment of the treatment unit’s mechanical isocenter with kV, MV, and CBCT imaging systems. Its simple cubic design, featuring internal low-density spheres and fiducials, allows for rapid verification of spatial alignment and laser coincidence. An optional tilting base also enables evaluation of 6°-of-freedom couch motion.

In comparison, the RUBY phantom’s System QA insert provides similar daily isocenter and imaging alignment checks but with enhanced functionality. It incorporates bone-equivalent and high-density markers to emulate anatomical contrast and perform Winston–Lutz tests via an embedded central sphere. As a result, daily QA using RUBY includes verification of kV/MV image matching using clinical bone presets and clear fiducial visibility—an advantage over Penta-Guide’s acrylic/air construction, which lacks CBCT registration capability using bone presets.

Regarding ease of use, both RUBY and Penta-Guide benefit from compact design and straightforward daily setup. RUBY’s insert system is designed for rapid interchange—its “plug-and-measure” holders enable switching ion chambers, diodes, or films without needing to reassemble the phantom. In practice, changing inserts adds minimal time once staff are accustomed to the process. This versatility enhances its clinical utility, allowing one RUBY phantom to be used for monthly Winston–Lutz checks, routine IGRT/SGRT verifications, and patient-specific QA, potentially replacing or supplementing several single-purpose devices. The trade-off, however, is that users must be proficient with all of RUBY’s capabilities, whereas simpler tools (like MapCheck or Penta-Guide) offer a more narrowly focused, plug-and-play experience.

## Clinical integration and workflow implementation

Integrating the RUBY phantom into the clinic’s QA protocols necessitated coordinated changes to routine workflows, documentation, and staff training. A stepwise QA schedule was established, with RUBY assuming multiple roles. Each morning, therapists or physicists use the RUBY System QA insert similarly to the Penta-Guide. Positioned on the treatment couch—often indexed to fiducial markers or a frame for reproducibility—a series of checks are performed to verify the coincidence of room lasers, treatment isocenter, and imaging isocenters (kV and MV). In practice, this includes a Winston–Lutz-type test using orthogonal kV images (or MV/kV pairs) of the central sphere to confirm the radiation isocenter diameter remains within 1 mm. Weekly, the phantom’s laser alignment lines are used to assess couch pitch/roll by applying known 6D shifts with the optional tilting base and verifying correct system detection. These tests have been formally integrated into the departmental QA Standard Operating procedures (SOPs), helping detect systematic couch calibration drifts [30].

For stereotactic treatment plans (SRS/SRT), the department mandates independent QA for each plan. RUBY is incorporated as an optional but valuable tool in this process. Typically, the plan is delivered to the phantom following CBCT-based alignment, mirroring patient setup. A measured point dose, using a micro-ionization chamber, is then compared with the TPS-calculated dose. A ± 3% agreement threshold is maintained, aligning with clinical standards. In cases involving steep dose gradients (e.g., near bone or air interfaces), discrepancies prompted either repeated measurements with improved setup or replanning. Overall, RUBY’s integration into patient QA was seamless, with physicists noting the benefit of using the same phantom for both machine and plan QA, promoting consistency. For planar dose verification, the PTW SRS 1600 array is still used selectively, but RUBY allows simultaneous geometric and absolute dose verification. This aligns well with AAPM TG-101, which emphasizes the importance of both localization and dosimetric QA for SRS/SBRT.

Quarterly, we perform comprehensive end-to-end tests. A dummy stereotactic plan is created on the phantom’s CT dataset, which includes pre-delineated “targets.” The workflow spans imaging (CT simulation, optional MR imaging, and CT–MR fusion using MR-visible markers), planning (evaluating TPS dose calculation accuracy in heterogeneous media), and delivery (SGRT setup, IGRT verification, dose measurement). These simulations serve not only QA but also training purposes. New physicists and RTTs gain practical experience in executing stereotactic workflows under both ideal and error-induced conditions. This hands-on training enhances familiarity with advanced tools, such as 6D couches and SGRT systems.

## Limitations of the performed QA tests

Despite the comprehensive capabilities of the RUBY phantom, certain limitations must be acknowledged. In this study, stereotactic plan verifications were primarily based on point-dose measurements, which capture dose at a single location within a complex three-dimensional distribution. While this method provides high precision, it may not fully represent spatial dose variations [27]. The observed dose deviations, although well within the clinically acceptable ± 3% limit recommended by AAPM TG-119 [31] and TG-218 [20], consistently indicated a slight overestimation by the treatment planning system (TPS). This systematic positive bias was evident in both detectors used, with median dose differences of + 0.79% for the Semiflex 3D and + 0.60% for the Pinpoint 3D. Several factors may contribute to this trend. The finite sensitive volume of both chambers can cause volume averaging effects, especially in high-gradient regions, potentially leading to relative under-response of the detector when compared to TPS-calculated point doses. Even minimal inaccuracies in detector positioning within the phantom may result in measurements being taken at slightly offset locations, further skewing the dose comparison. Additionally, minor limitations in TPS beam modeling, heterogeneity corrections, or resolution, particularly in small fields, may also contribute to the observed dose discrepancies. Subtle mismatches between the physical phantom setup and the idealized geometry assumed by the TPS may further influence the results. Although these deviations remain within acceptable clinical tolerance, the consistent trend underscores the importance of adopting more comprehensive 3D fluence-based patient-specific QA strategies, especially when dealing with stereotactic or highly conformal treatments where spatial accuracy is critical. While film dosimetry provides some mitigation by offering two-dimensional dose verification, it remains labor-intensive and impractical for routine use. Consequently, subtle discrepancies in composite dose distributions, such as off-isocenter dose spill, may go unnoticed. To address this, the integration of 2D or 3D detector arrays is recommended as a complementary approach for robust patient-specific QA [32].

Second, the phantom’s setup introduces complexity. Though modular by design, correct assembly and alignment are crucial [25]. Improper seating of inserts or misalignment with the phantom’s coordinate system can introduce errors. Also, there is the possibility of inter-user variability during manual registration, which can be reported after a long-term study. During Winston–Lutz tests, accurate centering of the embedded sphere is essential. Even a 1 mm tilt or offset can result in failed isocenter tolerance checks, necessitating realignment and repeat imaging. In contrast, simpler phantoms like ball-bearing-on-stem designs offer a more streamlined setup for isocenter localization. RUBY’s imaging characteristics also pose challenges. Though equipped with bone-equivalent rods and MR-visible structures, our TPS’s auto-registration algorithms sometimes struggled with the phantom’s geometry. Because phantoms consist of artificial shapes and uniform materials, they lack the variability of real patient anatomy, limiting the realism of registration tests. A more anthropomorphic phantom might better emulate clinical fusion conditions.

Third limitation of this study is that, despite performing Winston-Lutz tests, gantry sag-related effects were not visualized, due to the limited sample size. Gantry sag is a well-documented phenomenon in C-arm linear accelerators, with the potential to introduce significant geometric and dosimetric uncertainties, particularly in high-precision treatments such as SRS and VMAT. Previous studies have demonstrated the magnitude and clinical relevance of gantry sag, highlighting the importance of its characterization and compensation [33–36]. Although assessing gantry sag was beyond the scope of the current work, its potential impact is essential to guide future investigations and emphasize the need for comprehensive mechanical quality assurance in stereotactic and arc-based treatments.

Furthermore, our QA did not evaluate dynamic delivery scenarios such as motion management, gating, or MLC tracking. All tests used static setups, limiting insight into system performance under motion. This is particularly relevant for frameless spine or lung SRT, where respiratory motion or intrafraction drift can impact accuracy. RUBY cannot simulate such motion or deformation. Similarly, beam gating and MLC tracking tests require dynamic phantoms or motion platforms, which were beyond this study’s scope. Finally, practical issues such as beam hardening and CT artifacts from internal rods or spheres could affect dose calculation if not properly modeled. Hence, while the RUBY phantom provides robust static geometric and dosimetric QA, it cannot fully substitute for dynamic motion or full 3D verification when clinically required.

## Future recommendations

Our clinical experience with the RUBY phantom highlights the value of combining versatility with practical usability in modern QA devices. Initially, the learning curve was notable—its modularity and insert-specific workflows demanded focused training and SOPs development. However, once staff became familiar, the phantom enabled consistent and reproducible QA across a wide range of tasks. Its integrated design reduced clutter and minimized errors associated with transitioning between different QA devices. One of the most appreciated features was the insert system’s plug-and-play nature [37, 38]. Swapping between CBCT alignment, isocenter verification, and patient QA inserts was quick and preserved positional accuracy. The phantom’s weight was manageable by a single user, and its flat base facilitated reproducible placement on indexed couch systems. Nonetheless, a few improvements could enhance the user experience further. First, compatibility with 2D or 3D detector arrays would allow for broader dosimetric sampling, particularly useful in complex multi-target SRS plans. Second, a dedicated mobile application or QR-linked manual for insert-specific guidance would support on-demand training, particularly useful during off-hours or for rotating staff [39].

Beyond QA, RUBY has emerged as a valuable training tool. Routine QA drills evolved into hands-on teaching sessions, where simulated errors (e.g., couch misalignment, image fusion errors) were intentionally introduced. This educational use reinforces the importance of end-to-end QA not only for equipment validation but also for staff readiness and procedural standardization.

## Conclusion

This study highlights the RUBY phantom as a clinically robust and versatile platform for comprehensive quality assurance (QA) in high-precision radiotherapy. Through systematic evaluation of its modular inserts, the phantom enabled validation of imaging systems, mechanical isocenter congruency, and dosimetric accuracy under clinically relevant conditions. CBCT- and MV/kV planar image-based positioning workflows demonstrated sub-millimetric alignment accuracy, confirmed via automated couch correction and verified with data analysis. Winston-Lutz testing across multiple gantry, collimator, and couch angles revealed maximal deviations of ≤ 0.4 mm, well within AAPM TG-142 thresholds. Point dose verifications for 61 SRS patients using PTW Semiflex 3D and PinPoint 3D chambers showed dose agreement within ± 3% of TPS-calculated values, underscoring the system’s dosimetric fidelity. End-to-end testing further validated the integrity of the complete radiotherapy chain, including multi-target stereotactic plans involving both coplanar and non-coplanar arrangements. Across all scenarios, the RUBY phantom maintained high reproducibility, geometric stability, and adaptability to evolving treatment modalities. These findings substantiate the phantom’s critical role in ensuring advanced radiotherapy’s safety, accuracy, and clinical efficacy. Its integration into routine QA protocols supports the delivery of precision treatments and aligns with the growing emphasis on outcome-driven, patient-centric radiotherapy approaches.

## Supplementary information

Supplementary material 1.

## Data Availability

No datasets were generated or analysed during the current study. The authors declare no competing interests.
