# Supplementary material for: Clinical implementation of a multifaceted quality assurance phantom for high-precision radiation therapy: an institutional experience
Source: J Egypt Natl Canc Inst. 2025 Sep 1;37:57. doi: 10.1186/s43046-025-00314-x (PMC13313522; doi:10.1186/s43046-025-00314-x)
Supplement: Supplementary file 1 — Supplementary material 1. [file 43046_2025_314_MOESM1_ESM.docx]

Supplementary File

**Table** shows the different parameters for End-to-end testing insert (Baseline and measured)
